# Predictive Value of Interferon γ Receptor Gene Polymorphisms for Hepatocellular Carcinoma Susceptibility

**DOI:** 10.31557/APJCP.2021.22.6.1821

**Published:** 2021-06

**Authors:** Salah Aref, Aymen Zaki, Essam Mostafa El Mahdi, Eman Adel, Monier Bahgat, Enas Gouda

**Affiliations:** 1 *Department of Clinical Pathology, Haematology Unit, Mansoura University Oncology Centre, Mansoura University, Egypt. *; 2 *Mansoura University Oncology Center Laboratories; Mansoura University; Egypt. *; 3 *Department of Internal Medicine, Mansoura Faculty of Medicine, Mansoura University, Egypt. *

**Keywords:** Cirrhosis, HCC, interferon gamma receptor, polymorphism

## Abstract

**Background:**

Recent reports suggested relation between Interferon Gamma (IFN-γ) gene polymorphism and the risk of development of HCC on top of hepatic cirrhosis. The aim of this study was to address the predictive value of Interferon Gamma gene receptor (IFN-γR) polymorphisms for the occurrence of hepatocellular carcinoma on top of liver cirrhosis.

**Patients and Methods:**

This is a case control study performed on patients selected from the outpatient hepatology clinic, specialized medical hospital, Mansoura University, Egypt, from August 2017 to February 2019. The included patients were categorized into two groups; 60 patients with HCC on top of cirrhosis and 20 patients with hepatic cirrhosis. For all patients IFN-γR polymorphism was identified by RFLP.

**Results:**

Our study showed that HCC patients had male predominance. Additionally, diabetes mellitus (DM) was found in 28.3% of total HCC patients. Half of HCC patients in this study were from rural areas (50%). The frequency of AA at position -611 in the IFN-γR (-611 IFN-γR) was significantly higher in the HCC group as compared to cirrhotic group (P=0.021). Moreover; the frequency of CC and CT genotypes of IFN-γR -56 was not significantly different in HCC group as compared to control group (P>0.05). The IFN-γR (-611 IFN-γ) AA genotype significantly increased risk of HCC (OR= 0.78, 95% CI= 0.10-6.39; P= 0.042).

**Conclusion:**

The analysis of IFN-γR -611 single nucleotide gene polymorphism could be a valuable marker for predicting subgroup of cirrhotic patients with high risk of developing HCC. Cirrhotic patients have AA genotype of IFN-γR-611 recommended to be under close follow up.

## Introduction

Hepatocellular carcinoma (HCC) is the most common primary liver malignancy accounts for 70-90% of all primary liver cancer worldwide. It is the sixth most common cancer worldwide and the third most common cause of cancer death (Dai et al., 2014). Also, HCC is the most common cause of death in patients with chronic hepatitis C virus (HCV) infection and responsible for nearly one million deaths each year (Andrade et al., 2009). HCC is the main complication of cirrhosis, and shows a great incidence in Egypt, which may be the result of hepatitis B virus (HBV) and HCV as primary risk factors (Gomaa et al., 2014). In Egypt, the burden of HCC has been increasing with doubling the incidence rate in the past 10 years (Rashed et al., 2020). It represents 14.8% of all cancer mortality in Egypt (Rashed et al., 2020). HCV patients with cirrhosis are at risk of hepatocellular carcinoma (HCC) development with an annual rate of ≈ 3.5% (Aljabban et al., 2020). The carcinogenesis of HCV-associated HCC is a multistep process involving upregulation of inflammatory cytokines and induction of oxidative stress from chronic hepatitis, fibrosis, liver regeneration, and finally, development of cirrhosis (Liang and Heller, 2004). At the present time, HCC is increasingly recognized at a much earlier stage as a result of the routine screening of patients with known cirrhosis using cross-sectional imaging studies and serum alpha-fetoprotein (AFP) measurements (Alison et al., 2005). But, the sensitivity of AFP for diagnosis of HCC is 41–65% and specificity is in the range of 80–95%, when an AFP cut-off of 20 ng/mL is used (Daniele et al., 2004); about 50% of patients with HCC have AFP lower than 20 ng/mL. So, AFP cannot be used as the sole tool for HCC surveillance as it is associated with significant false-positive results related to hepatic activities (Farinati et al., 2006).

Abdominal ultrasonography is the most common imaging method for HCC surveillance because it is simple, non-invasive, inexpensive and allows a real-time observation. But, the evaluation of actual sensitivity of ultrasound is difficult due to lack of definite good standards for HCC (Coli et al., 2006). So, combined use of AFP and ultrasound can increase the rate of HCC detection, but it also increases the costs and the false-positive rate from 2.9% (ultrasound alone) or 5.0% (AFP alone) to 7.5% (combined) (Zhang and Yang, 1999). That’s why the need for more sensitive and specific tool has emerged for early diagnosis of HCC. Interferons (INFs) are group of proteins released by host cells in response to several pathogens such as viruses, bacteria, parasites and tumor cells. IFNs belong to large class of proteins known as cytokines, molecules used for communication between cells to trigger the protective defenses of the immune system that help in eradicating pathogens (Cohen and Parkin, 2001). Interferon gamma (IFN-γ) is a cytokine that is the only member of type II class of interferons which is critical for innate and adaptive immunity. Its importance in immunity arises from its ability to interfere with viral replication and more importantly its immune-stimulatory and immune-modulatory effects and is produced predominantly by natural killer (NK) and natural killer T (NKT) cells as part of the innate immune response, and by CD4 Th1 and CD8 cytotoxic T lymphocyte (CTL) effector T cells once antigen-specific immunity develops (Schoenborn and Wilson, 2007). One series that enrolled 63 HCC patients and 20 HBV carriers showed that serum IFN-γ level correlates with tumor status and clinical outcome. Serum IFN-γ level may reflect the host antitumor immunity in HCC and may be a potential marker to predict clinical outcome following curative treatment of HCC (Cheng et al., 2013). In this work, we are going to compare Interferon Gamma receptor (IFN-γR) polymorphism genotypes in HCV-related cirrhosis patients with and without hepatocellular carcinoma.

The aim of this work was to study of IFN-γR polymorphism as predictive biomarkers for cirrhosis patients who are at high risk for transformation to hepatocellular carcinoma.

## Materials and Methods

*Patients and Methods*

A case control study was performed on patients selected from the outpatient Hepatology and HCC early detection clinic, specialized medical hospital (SMH), Mansoura University, Egypt, from August 2017 to February 2019. All patients were enrolled in our study after signing an informed consent. Approval by the medical ethics research committee, Faculty of Medicine, Mansoura University was obtained.

*Study groups*

*Participants divided into 2 groups:*

*Group I:*

Sixty cirrhotic patients with HCC proved radiologically by abdominal US and triphasic abdominal CT. Similarly, this group was further subdivided according to CTP (Child-Turcott-Pugh) scores into 3 subgroups; Child A, B, and C. Also, BCLC (Barcelona Clinic Liver Cancer) scoring system was adopted for classifying HCC cases. Additionally, Milan and up-to-seven criteria (new Milan criteria) were considered in HCC patients.

*Group II:*

This group included twenty cirrhotic patients without HCC. The cirrhotic group was further subdivided according to CTP score into 3 subgroups; Child A, B, and C.

*Exclusion criteria for selected patients*

Patients with concomitant or past history of cancer, HCC patients who previously received specific treatment, patients with history of recent surgery, patients with renal impairment, presence of severe co-morbidity as advanced renal failure, decompensated heart failure; and patients with any inflammatory conditions as spontaneous bacterial peritonitis (SBP) or chest infection.

*Sample size*

Sample size was calculated using Online Sample Size Estimator (OSSE) for case-control SNP study. The web address (http://osse.bii.a-star.edu.sg/calculation1.php) was accessed on 21-1-2017 to calculate the sample size of a large-scale study. 

*Methods*

*All subjects were subjected to*

*Detailed medical history*

Personal history (including special habits; smoking, alcoholics or drug addiction).

The complaint and reason of attendance.

History of present illness, presence of comorbidities like diabetes mellitus (DM) or hypertension (HTN).

Past history of admission to any hospital for any cirrhosis related complication like hepatic encephalopathy, gastrointestinal bleeding, etc.

*Full clinical evaluation including*

Manifestations of liver cell failure and portal hypertension (like jaundice, ascites, ecchymosis, palmar erythema, lower limb edema).

*Laboratory tests including*

1- Biochemical tests

Liver function tests: by auto-analyzer Roche Cobas Integra-800 Serum albumin (gm/dl), bilirubin (mg/dl), hepatic enzymes [AST (Aspartate Amino Transferase) (U/l), ALT (Alanine Amino Transferase) (U/l)]. Renal function tests: Serum creatinine by Roche Cobas Integra -300.

2- Bleeding profile: PT (prothrombin time in seconds) and INR (International Normalized Ratio) by auto-analyzer Sysmex, Japan.

3- Complete blood count (CBC): by automated hematology analyzer Cell dyne 1700. Hemoglobin ―Hb‖ (gm/dl), red blood cell count ―RBC (103/cmm), white blood cell count ―WBC‖ (103/cmm), platelet count (103/cmm).

4- Virology markers:

Murex anti HCV ELISA for HCV.

Murex HBV surface Ag ELISA for HBV.

5- Biomarkers:

- Alpha-fetoprotein (AFP) level was measured on the Elecsys 2010 using electro- chemiluminescence immunoassay (ECLIA) for the in vitro quantitative determination of AFP in human serum and plasma.

6- Interferon Gamma (IFN-γ) gene Polymorphisms

Method of Interferon Gamma (IFN-γ) gene Polymorphisms:

The method used to determine polymorphism includes DNA extraction/ Polymerase chain reaction (PCR)/ Restriction fragment length polymorphism (RFLP). The primers used for amplification are For SNP-56 Forward: TGCATGACAAGGGGTAGGAG Reverse: CAACCAGGTGAAGTCCAAGAG For SNP-611 Forward: CTCTTCATGAGAGGCTGTCT Reverse: TAACTCTTGGAGTTCACCTGG Two restriction enzymes used to detect IFN-γR polymorphisms according to the SNP position. AfeI and Hpy188I (New England Biolabs, USA) used to detect genotyping for SNP-56 and SNP-611 respectively.

*Statistical Analysis*

Data were entered and analyzed using SNPStats software. Predictors for IFN-γR polymorphisms were assessed by multivariate logistic regression analysis. Multivariate analysis included all significant parameters from nonparametric univariate analysis. Differences between groups were assessed by χ2 test or Fisher-Freeman-Halton’s test, Kruskal-Wallis test, and Wilcoxon-Mann-Whitney-U-test as appropriate. All tests were two-sided and p-values below 5% were considered significant.

## Results

*Clinical and Laboratory findings in studied groups*

This study involved 80 patients divided into two groups: Group 1: HCC group (n=60). Group 2: Cirrhosis group (n=20). The clinicodemographic data in the two groups are shown in Table 1. Table 1 shows a statistically significant older age, higher frequency of portal hypertension and moderate splenomegaly in the HCC group as compared to the cirrhosis group. Smoking was more prevalent in cirrhotic patients with HCC. HCC patients were more clinically silent at time of presentation and were discovered during routine follow up. Ascites, jaundice and abdominal pain were the commonest presentations in cirrhotic patients with HCC while lower limb edema was the commonest presentation in cirrhotic patients without HCC. Moreover; this Table shows a statistically significantly higher MELD score in HCC group vs. cirrhosis group. CTP score was higher also in HCC group but it didn’t achieve a statistical significance. 

The laboratory data showed statistically significantly higher values of ALT, AST, INR, S. bilirubin and AFP in the HCC group as compared to the cirrhosis group (Table 2).

*Association of FN-γR genotypes with Cirrhotic and HCC*

Table 3 shows a statistically significantly higher frequency of A allele in the HCC group as compared to cirrhotic group while G allele is statistically significantly higher in the cirrhosis group as compared to HCC group (P=0.042). In contrast; there is no significant association between IFN-γR1 -56 genotypes and cirrhotic or HCC (Table 4).

*Odds ratio (OR) shows the relationship between IFN-γR -611 and HCC development on top of cirrhosis*

The logistic regression analysis was done in order to predict the impact of IFN-γR -611 polymorphism on the risk of HCC transformation on top of cirrhosis. The analysis revealed that the odds ratio of AA allele on cirrhosis transformation into HCC is OR= 0.78 (95% CI = 0.10-6.39; P= 0.041) (Table 5).

*OR shows the relationship between IFN-γR -56 genotypes and HCC development on top of cirrhosis*

The logistic regression analysis revealed that SNP of Interferon-56 Gamma receptor gene odds ratio was OR = 0.67 (95% CI = 0.03-16.42; P>0.05) (Table 6).

**Table 1:** Clinico-Demographic Data in the Two Studied Patients Groups

	Group		P value
	HCC (n=60)	Cirrhosis (n=20)	
Age (years):	60.80±8.845	47.85±13.056	<0.01
Sex Frequency (%):			
Male	42 (70.0%)	12 (60%)	>0.05
Female	18 (30.0%)	8 (40%)	
Residency:			>0.05
Rural	30.0 (50.0%)	11 (55%)	
Urban	30.0 (50.0%)	9 (45%)	
Smoking			>0.05
Non smoker	25 (41.7%)	10 (50.0%)	
Ex-smoker	21 (35.0%)	6 (30.0%)	
Current smoker	14 (23.3%)	4 (20.0%)	
Diabetes, Yes	17 (28.3%)	(20.0%)4	>0.05
Hypertension, Yes	9 (15.0%)	(10.0%)2	>0.05
Renal impairment, Yes	4 (6.7%)	(5.0%)1	>0.05
Ascites			
No	27 (45.0%)	12 (60.0%)	>0.05
Mild	24 (40.0%)	4 (20.0%)	
Moderate to sever	9 (15.0%)	4 (20.0%)	
Hepatic encephalopathy			>0.05
No	54 (90.0%)	19 (95.0%)	
Grade 1 or 2	6 (10%)	1 (5.0%)	
Jaundice, Yes	29 (48.3%)	6 (30.0%)	>0.05
Abdominal pain, Yes	25 (41.7%)	5 (25.0%)	>0.05
Lower limb edema, Yes	15 (25.0%)	8 (40.0%)	0.199
History of variceal bleeding, Yes	17 (28.3%)	4 (20%)	0.463
Portal hypertension, Yes	42 (70.0%)	8 (40.0%)	0.016
Splenomegaly			
Mild	14 (23.3%)	12 (60%)	0.002
Moderate	14		

**Table 2:** Laboratory Data of the Studied Patients Groups

Parameters	Patients group			Test of significance
	HCC (n=60)	Cirrhosis (n=20)	t / χ2	P Value
Hemoglobin (g/dL)	10.5 (9.3-12.27)	11.5 (8.7-12.0)	-0.122	>0.05
WBCs (/cmm^3^)	4.8 (2.6-7.0)	3.8 (2.0-6.27)	-1.239	>0.05
Platelets count (/cmm^3^)	108.5 (74.5-148.0)	174 (62.0-275.5)	-1.617	>0.05
ALT(U/L)	57.5 (40.5-172.25)	36.5 (24.0-53.25)	-3.523	<0.01
AST(U/L)	38 (27.25-60.75)	25.5 (15.5-32.75)	-3.413	0.01
S.Creatinine (mg/dL)	1.1 (0.72-1.60)	0.90 (0.72-1.27)	-1.022	>0.05
S. albumin (g/dL)	2.95 (2.4-3.3)	3.6 (2.15-4.75)	-1.607	>0.05
S. bilirubin (mg/dL)	1.9 (1.0-4.40)	0.8 (0.6-2.7)	-2.72	0.01
INR	1.3 (1.2-1.57)	0.1 (1.02-1.45)	-2.316	0.02
AFP ng/ml	60.8 (12.87-430.25)	3.4 (2.42-9.7)	-4.756	0.01

**Table 3:** SNP of IFN-γR-611 Genotypes Frequency among Studied Patients Groups

	HCC (n=60)	Cirrhosis (n=20)	P value
AA	34 (56.7%)	4 (20%)	0.026
AG	20 (%33.3)	13 (65%)	
GG	6 (10%)	3 (15%)	

P value, 0.026 (Monte Carlo significance)

**Table 4:** SNP of IFN-γR -56 Receptor Genotypes Frequency among Studied Groups

	HCC (n=60)	Cirrhosis (n=20)	P value
CC	8	21	>0.05
CT	8	21	
TT	4	18	

t / χ2, 7.410; P value, 0.026 (Monte Carlo significance).

**Table 5:** Odds Ratios (OR) of IFN-γR -611 Genotypes for HCC Development on Top of Cirrhosis

	Cirrhosis (n=20)	HCC (n=60)	OR (95% CI)	P-value
AA	5 (25.0%)	34 (56.7%)	0.29 (0.08-1.11)	
AG	13 (65.0%)	20 (33.3%)		0.042
GG	2 (10.0%)	6 (10.0%)		

**Table 6:** OR of IFN-γR -56 Genotypes for HCC Development on Top of Cirrhosis

Genotype	Cirrhosis (n=20)	HCC (n=60)	OR (95% CI))	P value
CC	8	21	0.51(0.03-7.25)	>0.05
CT	8	21		
TT	4	18		

## Discussion

In this study diabetes mellitus was identified in 28.3% of total HCC patients. This finding is higher than the figure reported by Abdel-Wahab et al., (2007) which was 13.6%. This discrepancy could be attributed to the increasing prevalence of DM among Egyptians. The prevalence of DM was 15% in 2015 versus 8% in 2007 (Sherif and Sumpio, 2015). Previous study stated that there is positive correlation between the history of diabetes mellitus and HCC (Lagiou et al., 2000).

Half of HCC patients in this study were from rural areas. Previous reports (Abdel-Wahab et al., (2007) and Shaker et al., (2013) found low percentage of patients from rural areas among HCC group. This may be explained by increased urbanization among Egyptians. The frequency of patients with positive smoking history among our HCC patients group was 58.3%. This figure is matched with that detected by Koh et al., (2011) who considered that smoking is an independent risk factor for HCC. 

In the current, the MELD score was detected in HCC patients group in a significant high figure as compared to cirrhotic group. Likewise, CTP score was detected in HCC group in a high figure as compared to cirrhotic group but the difference did not reach the level of significance.

HCC incidence is rising in areas with high prevalence of HCV or HBV infection. In general, almost all HCC cases are preceded by chronic hepatitis or liver cirrhosis, which are mainly caused by hepatitis C, hepatitis B and alcoholic liver disease. Egypt is considered to have the highest prevalence of HCV worldwide, estimated to be around 14% of general population (Omar et al., 2013) and up to 90% of HCC cases were attributed to HCV infection (Ezzat et al., 2005). Early detection of HCC on top of cirrhosis is very important to start therapy early (Kelly and Bird., 2011). Surveillance guidelines for detecting HCC had been subjected to continual changes regarding the protocol and tools used. The standard surveillance protocol depends on two non-ideal tools; ultrasonography and AFP. However, the sensitivity of ultrasound for detecting HCC in cirrhosis is 58% only (Gambarin-Gelwan et al., 2000). 

In this study, two polymorphisms of IFN-γR promoter were identified, including two SNPs at position -56 and -611. Analysis of IFN-γ R promoter alleles and genotypes distributions in the two studied groups revealed a significant association between IFN-γR promoter polymorphisms and the susceptibility to HCC. Based on this polymorphism at position -611 in the IFN-γ gene (-611 IFN-γ), three genotypes were possible; A/A, A/G, G/G. A/A genotype was frequently detected in the HCC group as compared to cirrhotic group. While, A/G genotype was frequently detectedfounded in the cirrhosis group. 

Interferon Gamma (IFN-γ) is a soluble cytokine and is a type II class of interferons (Gray PW et al., 1982). IFN-γ plays a vital role in inducing and modulating a variety of immune responses and is secreted in response to infection (Schroder et al., 2004). IFN-γ has antiviral, immuno-regulatory and antitumor functions (Schroder et al., 2004) as it produces a range of physiological and cellular actions through alteration of transcription in up to 30 genes. It is also identified to have a protective role against tumor development and cancer immuno-editing (Ikeda et al., 2002 ; Horras et al., 2011). Unfortunately, there are few studies about its diagnostic validity for HCC. To address this point, we tried to detect the diagnostic role of IFN-γR as a tumor marker for HCC in our study.

Among the studied IFN-γR promoter polymorphisms (-611 IFN-γ), the AA genotypes was frequently detected in HCC patients as compared to other genotypes (AG, GG). Previous report suggests that the genetic changes in the setting of chronic HCV infection predispose patients to developing HCC (Aljabban et al., 2020). On other hand; another study stated that there is no strong evidence supporting a statistically significant association between IFN-γ rs2430561 polymorphism and HCC susceptibility (Sun et al., 2015). In contrary; Interferon alpha promoter receptor polymorphism has no risk of HCC transformation on top of hepatitis B virus infection (Klenerman et al., 2018).

The IFN-γR genetic polymorphism at -56 analysis reveal that there are three genotypes were possible; C/C, C/T, T/T. Our study revealed that C/C and C/T genotypes frequencies were insignificantly increased in the HCC group. While T/T genotype was detected in a high frequency among cirrhotic group. 

The polymorphisms in the regulatory regions and introns of IFN-γ and its receptor genes can change the expression of IFN-γ in patients with acute HBV infection and chronic HBV carriers. This means that polymorphisms in the IFN-γ and its receptor genes can increase the risk of hepatitis developing ( Rizvi et al., 2012).

The relation between AA IFN-γR genotype and increased risk of HCC transformation on top of cirrhosis could be attributed to down regulation of the signaling pathway of IFN-γ. This will lead to decrease its biological effects as anticancer cytokine. Increased IFN-γ serum expression has been reported to promote antitumor activity, whereas sustained low-level expression of IFN-γ triggers tumorigenesis (He et al., 2005). 

In conclusion, the analysis of IFN-γR-611 single nucleotide gene polymorphism (A/A genotype) could be a valuable marker to define subgroup of patients who at high risk of transformation to HCC in HCV related cirrhotic patients.

## Author Contribution Statement

Conceptualization: Salah Aref. Data curation: Aymen Zaki; Essam Mostafa El mahdi. Formal analysis: Monier Bahgat; Aymen Zaki; Essam Mostafa El mahdi. Investigation: Monier Bahgat; Enas Gouda. Methodology: Eman Adel; Enas Gouda. Resources: Aymen Zaki. Writing – original draft: Salah Aref; Enas Gouda. Writing – review & editing: Salah Aref.
